# Recommendations for wider adoption of clinical pharmacy in Central and Eastern Europe in order to optimise pharmacotherapy and improve patient outcomes

**DOI:** 10.3389/fphar.2023.1244151

**Published:** 2023-08-02

**Authors:** Kamila Urbańczyk, Sonja Guntschnig, Vasilis Antoniadis, Slaven Falamic, Tijana Kovacevic, Marta Kurczewska-Michalak, Branislava Miljković, Anna Olearova, Inese Sviestina, Attila Szucs, Konstantin Tachkov, Zita Tiszai, Daisy Volmer, Anna Wiela-Hojeńska, Daniela Fialova, Jiri Vlcek, Matej Stuhec, Anita Hogg, Michael Scott, Derek Stewart, Alpana Mair, Silvia Ravera, François-Xavier Lery, Przemysław Kardas

**Affiliations:** ^1^ Department of Clinical Pharmacology, Wroclaw Medical University, Wroclaw, Poland; ^2^ Regional Specialist Hospital in Wroclaw, Wroclaw, Poland; ^3^ Tauernklinikum Zell am See, Zell am See, Austria; ^4^ School of Pharmacy and Pharmaceutical Sciences, Ulster University, Coleraine, Northern Ireland; ^5^ Independent Expert, Thessaloniki, Greece; ^6^ Faculty of Medicine Osijek, Josip Juraj Strossmayer University of Osijek, Osijek, Croatia; ^7^ Pharmacy Department, University Clinical Centre of the Republic of Srpska, Banja Luka, Bosnia and Herzegovina; ^8^ Department of Pharmacokinetics and Clinical Pharmacy, Faculty of Medicine, University of Banja Luka, Banja Luka, Bosnia and Herzegovina; ^9^ Department of Family Medicine, Medical University of Lodz, Lodz, Poland; ^10^ Department of Pharmacokinetics and Clinical Pharmacy, Faculty of Pharmacy, University of Belgrade, Belgrade, Serbia; ^11^ Department of Clinical Pharmacology, University Hospital Bratislava—Hospital Ruzinov, Bratislava, Slovakia; ^12^ Faculty of Medicine, University of Latvia, Riga, Latvia; ^13^ Children’s Clinical University Hospital, Riga, Latvia; ^14^ Pharmacy Department, National Institute of Oncology, Budapest, Hungary; ^15^ Department of Organization and Economy of Pharmacy, Faculty of Pharmacy, Medical University-Sofia, Sofia, Bulgaria; ^16^ Department of Hospital Pharmacy, Bajcsy-Zsilinszky Hospital, Budapest, Hungary; ^17^ Institute of Pharmacy, Faculty of Medicine, University of Tartu, Tartu, Estonia; ^18^ Department of Clinical and Social Pharmacy, Faculty of Pharmacy in Hradec Králové, Charles University, Hradec Králové, Czechia; ^19^ Department of Geriatrics and Gerontology, First Faculty of Medicine in Prague, Charles University, Prague, Czechia; ^20^ Clinical Pharmacy Department, Hospital Pharmacy, Teaching Hospital Hradec Kralove, Hradec Králové, Czechia; ^21^ Department of Pharmacology, Faculty of Medicine Maribor, University of Maribor, Maribor, Slovenia; ^22^ Department of Clinical Pharmacy, Ormoz Psychiatric Hospital, Ormoz, Slovenia; ^23^ Medicines Optimisation Innovation Centre, Antrim Hospital, Antrim, United Kingdom; ^24^ College of Pharmacy, QU Health, Qatar University, Doha, Qatar; ^25^ European Society of Clinical Pharmacy, Leiden, Netherlands; ^26^ Effective Prescribing and Therapeutics, Health and Social Care Directorate, Scottish Government, Edinburgh, United Kingdom; ^27^ European Directorate for the Quality of Medicines & Healthcare, Council of Europe, Strasbourg, France

**Keywords:** clinical pharmacy, cost-effective treatment, medication errors, drug utilization, drug safety, medication adherence, health policy, polypharmacy

## Abstract

Clinical pharmacy as an area of practice, education and research started developing around the 1960s when pharmacists across the globe gradually identified the need to focus more on ensuring the appropriate use of medicines to improve patient outcomes rather than being engaged in manufacturing and supply. Since that time numerous studies have shown the positive impact of clinical pharmacy services (CPS). The need for wider adoption of CPS worldwide becomes urgent, as the global population ages, and the prevalence of polypharmacy as well as shortage of healthcare professionals is rising. At the same time, there is great pressure to provide both high-quality and cost-effective health services. All these challenges urgently require the adoption of a new paradigm of healthcare system architecture. One of the most appropriate answers to these challenges is to increase the utilization of the potential of highly educated and skilled professionals widely available in these countries, i.e., pharmacists, who are well positioned to prevent and manage drug-related problems together with ensuring safe and effective use of medications with further care relating to medication adherence. Unfortunately, CPS are still underdeveloped and underutilized in some parts of Europe, namely, in most of the Central and Eastern European (CEE) countries. This paper reviews current situation of CPS development in CEE countries and the prospects for the future of CPS in that region.

## 1 Introduction

The most recent definition formulated by the European Society of Clinical Pharmacy (ESCP) states: “Clinical pharmacy aims to optimize the utilization of medicines through practice and research in order to achieve person-centred and public health goals” (Dreischulte et al., 2022). This area of practice, education and research started developing around the 1960s when pharmacists across the globe gradually identified the need to focus more on ensuring the appropriate use of medicines to improve patient outcomes rather than being engaged solely in manufacturing and supply (Carter, 2016). Since that time numerous studies have shown the positive impact of clinical pharmacy services (CPS). CPS has been demonstrated to be economically beneficial in multiple evaluations throughout the years (Byrne and Dalton, 2017), including American College of Clinical Pharmacy summaries from 1996 onwards (Schumock et al., 1996; 2003; Perez et al., 2008; Touchette et al., 2014). An intensified involvement of the clinical pharmacist in all stages of the patient journey named “integrated medicines management” or “seamless care” has been demonstrated beneficial for both patients and healthcare systems due to reduced average length of stay in hospitals, reduced number of and longer time to readmissions, number of outpatients visits, medication burden and healthcare costs (Spehar et al., 2005; Scullin et al., 2007). The implementation of clinical pharmacy services leads to optimized drug utilization and improved drug safety (McMullin et al., 1999; Anderson and Schumock, 2009; Simoens et al., 2011; Reardon et al., 2015; Rychlickova et al., 2016). CPS stimulate appropriate prescribing, promote the use of drugs with a higher Medicines Appropriateness Index (MAI) and a lower Medicines Administration Error rate (Hanlon and Schmader, 2013; Scott et al., 2015). Pharmacist-led chronic disease management leads to higher patient satisfaction and improves patient adherence (Schulz et al., 2019; McCarthy and Thomas Bateman, 2022). Clinical pharmacists educate patients and enhance patient health literacy, which contributes to a reduction of adverse drug reactions (ADRs) (Spehar et al., 2005; Simoens et al., 2011). Medicines reviews and patient education before hospital discharge improves outpatient drug safety by reducing potential adverse effects, drug-drug interactions and inappropriate overuse of medication. Clinical pharmacy practices ensure safe prescribing with numerous scientifically validated tools (Curtin et al., 2019), particularly important for populations at higher risks of adverse drug outcomes, e.g., for older patients, paediatric population, and patients with renal and hepatic failure. Finally, CPS are also perfectly well placed to address current global challenges, such as the World Health Organisation (WHO) Global action plan on antimicrobial resistance (WHO, 2015). Pharmacist-led Antimicrobial Stewardship programs have proved successful in reducing antibiotic consumption and overall hospital expenditures, ensuring prudent use of antimicrobial agents (Brink et al., 2016). Another important target for CPS is Medication without Harm, the third global patient safety challenge launched in 2017 by the WHO (WHO, 2017). It aimed to reduce the level of severe avoidable harm related to medication by 50% over 5 years globally. Assuming that medication errors, drug-drug interactions are avoidable and can be significantly reduced or indeed prevented by improving the system and practice of medication use, the use of CPS seems to be a natural solution. Additionally, integrated medicines management models of providing CPS also correspond with another key action area—transition of care.

Moreover, the need for wider adoption of CPS worldwide becomes urgent, as the global population ages. Ageing leads to a rising prevalence of chronic diseases, and related multimorbidity, which further increases the need for medicine use and often leads to polypharmacy (Kardas et al., 2013). Thus, due to the an increasing proportion of older, the prevalence of polypharmacy is rising. In 2015, approximately 5% of the population in OECD countries was aged 80 years and above, This percentage is expected to double by 2050 (OECD, 2021). Of note is that multimorbidity increases markedly with age—a Scottish study reported that multimorbidity was prevalent in as many as 81.5% of individuals aged 85 years and over, with a mean of 3.02 comorbidities (Mair et al., 2017). This increasing number of comorbidities is associated with frequent polypharmacy not only due to the need to treat simultaneously various different conditions but also because current treatment guidelines are based mostly on “non-geriatric evidence.” This complicates the optimal selection of multidrug regimens particularly in very old and frail patients. In relation to the economic impact, it is estimated that mismanaged polypharmacy contributes to 4% of the world’s total avoidable costs due to sub-optimal medicine use, with a total of US$18 billion, representing 0.3% of total global health expenditure, that could be avoided by appropriate polypharmacy management (Mair et al., 2017). Apart from that, increasing complexity of treatment simply requires team efforts to improve patient outcomes. Collaborative care models including pharmacist were already shown to be beneficial in different settings of care (Grimes et al., 2014; Hahn et al., 2018; Lee et al., 2019).

Another key component related to polypharmacy is non-adherence to prescribed medication. Non-adherence may either be intentional or non-intentional. Both of these cases become more prevalent with higher number of drugs being prescribed and it has been demonstrated that there is a correlation between non-adherence and the number of medicines being taken (Kardas et al., 2013). Research suggests that between 50% and 80% of patients with chronic health conditions may be non-adherent. Non-adherence has been estimated to be responsible for 48% of asthma deaths, an 80% increased risk of death in diabetes and a 3.8-fold increased risk of death following a heart attack (Mair et al., 2017).

All these challenges urgently require adoption of a new paradigm of healthcare system architecture. Clinical pharmacy offers an effective answer to many of these challenges, as illustrated by case examples in the United States, Canada and Australia, countries that already allowed clinically trained pharmacists to work effectively with other healthcare professionals in direct patient care, in various aspects of medicine management and in different settings of care. In Europe, the United Kingdom serves as another good practice example of a healthcare system integrating CPS in both inpatient and outpatient settings of care.

Scotland has developed one of the few well-organized polypharmacy management programs in Europe (Stewart et al., 2017). NHS Scotland Polypharmacy Guidance offers probably the most comprehensive guidance on a patient-centred approach to ensure safe and appropriate use of medicines in patients using polypharmacy (Scottish Government Polypharmacy Model of Care Group, 2018). In 2012, a 7-step person centred approach was introduced to consider appropriate polypharmacy in the context of multimorbidity. Initially, polypharmacy reviews were incentivized by enhanced service payments to general practitioners and later by inclusion in their Quality Outcome Framework (QOF) targets. Since 2013, with the policy driver of the Governments “Prescription For Excellence” additional funding has enabled pharmacists to work alongside general practitioners to support appropriate polypharmacy management (Mair et al., 2019). Through its work, in 2015, Scotland led and EU project called SIMPATHY engaging with 10 partners across 8 EU countries to determine how the management of polypharmacy could be achieved across the EU by 2030. This work came up with six key recommendations: use a systems approach that has multidisciplinary clinical and policy leadership; nurture a culture that encourages and prioritizes the safety and quality of prescribing; ensure that patients are integral to the decisions made about their medicines and are empowered and supported to do so; use data to drive change and measure outcomes; adopt an evidence-based approach with a bias toward action; utilize, develop, and share tools to support implementation (Mair and Fernandez-Llimos, 2017).

In Northern Ireland, integrated medicines management (IMM) program including both hospital and community sectors has been also set up. It involves a number of activities performed by clinical pharmacists: medication reconciliation in conjunction with patient monitoring, patient counselling, discharge prescription preparation and collaboration at the community–hospital interface. IMM resulted in significant improvements in the quality and safety of medicines yielding both patients’ health gains and substantial savings for the healthcare system (Scott et al., 2015). This concept was further adopted in some other European countries including Republic of Ireland, Norway and Sweden (Gillespie et al., 2009; Hellström et al., 2011; Galvin et al., 2013; Lea et al., 2020).

Europe-wide, the Council of Europe has defined through the work of its European Directorate for the Quality of Medicines & Healthcare (EDQM) a legal framework for its 39 European member states for the promotion and implementation of the concept of pharmaceutical care and related services in health systems at a national level. This framework is provided by Resolution CM/Res(2020)3 on the implementation of pharmaceutical care for the benefit of patients and health services, adopted in 2020 (Committee of Ministers Resolution, 2020). Among other things, the Resolution covers aspects of healthcare workforce and education, advocating for strengthening the role of suitably qualified pharmacists for performing pharmaceutical care and clinical pharmacy services (Committee of Ministers Resolution, 2020). Countries where CPS were introduced are currently reaping various benefits, with optimized drug utilization, enhanced drug safety and medication adherence, among the others, leading to better sustainability of their healthcare systems (Gillespie et al., 2009; Hellström et al., 2011; Galvin et al., 2013; Schulz et al., 2019; Lea et al., 2020; McCarthy and Thomas Bateman, 2022).

Unfortunately, CPS are still underdeveloped and underutilized in some parts of Europe, namely, in most of the Central and Eastern European (CEE) countries. Therefore, this review describes the current situation of CPS development in CEE countries and the prospects for the future of CPS in that region. The CEE countries are defined most often as EU member states which were part of the former Eastern bloc. However, the scope of this paper covers also some other countries belonging regionally to Central and East European region, i.e., Austria, one entity of Bosnia and Herzegovina (Republic of Srpska—RS), Bulgaria, Czech Republic, Croatia, Estonia, Greece, Hungary, Latvia, Poland, Serbia, Slovenia and Slovakia. For ease of reading, they will be collectively called CEE countries in the rest of the text. Data presented in this paper were obtained thanks to the expertise of representatives from listed countries and a review of available national data related to the topic (literature, legislation, statistics, etc.), valid as per January 2023. Moreover, the goal was to emphasise the need for wider adoption of CPS in CEE countries and formulate relevant recommendations and call for action.

## 2 Current scenario of clinical pharmacy in CEE countries

### 2.1 Specificity of CEE countries

Along with many historical reasons, a need to change the paradigm of the healthcare system, stimulate acceptance of the new roles of pharmacists, and for the implementation of the new services before the investment can be recouped may be identified as the most important barriers of adoption and development of clinical pharmacy in CEE countries. Consequently, the current level of development and use of CPS in most of the CEE countries is still low.

Table 1 presents the basic characteristics of analyzed CEE countries taking into account their population size, share of population over 65 years with predicted changes, gross domestic product (GDP) *per capita* and healthcare expenditure. According to OECD data from 2021 regarding healthcare expenditure *per capita*, all excluding Austria, were below the European average. Moreover, Estonia, Latvia, Poland and Slovakia experienced marginal increase in number of physicians and nurses per 1,000 population over past 20 years. As underlined in OECD report: “Overall, countries with higher health spending and higher numbers of health workers and other resources have better health outcomes, quality and access to care.” This links with fact that projected share of population over 65 years will be gradually increasing in all regions in the coming years (OECD, 2021). With increasing age, multimorbidity, polypharmacy and health services utilization becomes more prevalent. A recent analysis of a large European cohort found polypharmacy to be present in 32.1% of citizens aged over 65 years, on average, with Czech Republic with 39.9% being the highest across the studied countries (Midão et al., 2018). Prevalence of polypharmacy, calculated dispensing of five or more drugs for the whole Polish population, ranged in 2019 from 21.8% for January-June, to 22.4% for July-December. However, among those aged 65+, the relevant numbers were 62.3%, and 62.9%, respectively (Kardas et al., 2021). Research from Slovakia showed that among 459 nursing homes residents over 65 years, polypharmacy (use of ≥ 5 drugs by patient daily) was present in 83% of all patients (Jankyova et al., 2020). According to data from Slovenia, 10% of the Slovenian population was treated with at least five medications concomitantly and 4% with over ten medications (Stuhec, 2021).

**TABLE 1:** Basic characteristics of analysed countries.

Country	Approximate population size (mln)^a^	Share of population over 65 (%) in 2019^b^	Share of population over 65 (%) prognosed for 2050^b^	GDP *per capita* USD PPP^c^	Healthcare expenditure *per capita* USD PPP^d^
1. Austria	8.9	19	28	59,976	5,705
2. Entity of Bosnia and Herzegovina—Republic of Srpska	1.1	N/A	N/A	6,699	1,477
3. Bulgaria	6.9	N/A	N/A	26,793	2,123
4. Croatia	3.9	N/A	N/A	34,023	2,079
5. Czechia	10.5	20	30	44,802	3,805
6. Estonia	1.3	20	28	43,494	2,507
7. Greece	10.6	22	35	31,177	2,319
8. Hungary	9.7	19	27	36,687	2,170
9. Latvia	1.9	20	32	35,150	2,074
10. Poland	38.2	18	30	37,771	2,289
11. Serbia	6.7	N/A	N/A	21,588	1,686
12. Slovakia	5.4	16	29	31,320	2,189
13. Slovenia	2.1	20	31	43,970	3,737

GDP, gross domestic product; PPP, purchase power parity.

^a^ Population size for 1, 3, 4, 5, 6, 7, 8, 9,10, 12, 13 (OECED, 2023c), for 2 (Institute of Statistics Republic of Srpska, 2023a), for 6 (Study programme on clinical, 1960).

^b^ Share of population over 65 for 1, 5, 6, 7, 8, 9, 10, 12, 13 (OECD, 2021).

^c^ GDP, *per capita* for all, except 2 (OECED, 2023a), for 2 (Institute of Statistics Republic of Srpska, 2023b).

^d^ Healthcare expenditure for 1, 6, 7, 8, 9,10, 12, 13 (OECD, 2021), for 3, 4, 5 (OECED, 2023b), for 2 (WHO, 2022), for 11 (WHO, 2018).

Nevertheless, CEE countries adopted to a lesser extent some of the tools designed to recognize potentially inappropriate prescribing like EU (7)-PIM list as reported by Fialová et al. (2019).

Similarly, the problem of medication non-adherence seems to be particularly pronounced in CEE countries: in a multinational study as many as 57.6% of Polish patients studied and 70.3% of Hungarian ones reported non-adherence to chronic therapy, whereas corresponding values for West European countries ranged from 24.1% for the Netherlands to 41.5% for England (Morrison et al., 2015).

All these new targets for healthcare systems need to be interpreted in the context of an increasing shortage of healthcare professionals in many European countries, not only in the CEE ones. These deficits are particularly problematic among nurses andphysicians, and the prospects for the future are even more concerning (OECD, 2021).

As illustrated above, there are many reasons to conclude that CEE countries urgently need to adopt new solutions aiding the provision of better healthcare for their citizens. One of the most appropriate answers to these challenges is to increase the utilization of the potential of highly educated and skilled professionals widely available in these countries, i.e., pharmacists, who can be engaged in direct patient care under the auspices of clinical pharmacy to provide better and faster access to healthcare.

### 2.2 Best practice examples among CEE countries

Although the majority of CEE countries are still lacking sufficient clinical pharmacist development, the Czech Republic and Slovenia can serve as best practice examples with their successful implementation of reimbursed CPS.

#### 2.2.1 Czech Republic

In the Czech Republic (CZ) postgraduate training in clinical pharmacy started back in 1981, with a long tradition of pregraduate education (since 1970’s). Speciality training in CP was always considered a core for the clinical growth of pharmacists into independent clinical experts skilled in individualization and optimization of drug schemes in high-risk patients and/or high-risk clinical situations.

At the beginning of specialty training in CZ, the first tutors were trained at departments of clinical pharmacology or abroad-in the United States, United Kingdom and other countries. Later, some tutors were employed in various clinical medical wards and some in hospital pharmacies with limited capacity to provide CPS in clinical wards. In 2005, under the Institute for Postgraduate Education in Healthcare in Prague, Department of Clinical Pharmacy, the first team of advanced clinical pharmacy tutors was established and each of the tutors was responsible for the development of a specific CP subarea (e.g., CP in palliative care, cardiology, oncology and hemato-oncology, geriatrics, pediatrics, etc.). Despite good quality specialty training and skilled supervisors, the lack of paid positions greatly limited further development of CPS. Moreover, hospital pharmacies employing the majority of graduates and specialists in clinical pharmacy engaged them mainly in duties within the pharmacy departments with limited access to clinical wards. Currently, specialty training in clinical pharmacy lasts 5 years and includes clinical rotations of CP trainees among various clinical inpatient and outpatient departments/healthcare facilities to enable the growth of CP specialists in all general aspects of CPS in various patient populations (Institute for Postgraduate Education in Healthcare, 2022) Higher level certified courses (above this general training) are planned, particularly for some specific areas of CP (e.g., CP in intensive care units, psychiatric departments and therapeutic drug monitoring for high-risk patient groups). Currently, not only the Institute for Postgraduate Education in Prague, but also the University Educational Centre in Clinical Pharmacy at the Faculty of Pharmacy, Charles University help with postgraduate education in clinical pharmacy. Specialty training is fully led on accredited CP workplaces (Faculty of Pharmacy in Hradec Kralove, 2023; Institute for Postgraduate Education in Healthcare, 2022).

According to “Concepts of Clinical Pharmacy field” (summarizing history, major guidelines and position of clinical pharmacy field) issued by both Czech professional clinical pharmacy societies—Section of Clinical Pharmacy of the Czech Pharmaceutical Society (Vlček et al., 2016) and Czech Professional Society of Clinical Pharmacy (Gregorová et al., 2014), stage 3 of medication reviews (comprehensive clinical medication reviews) can be independently provided by and remunerated (in inpatient and outpatient care) only for specialists in clinical pharmacy.

It is important to emphasize that after decades of intensive educational effort, the path to fully established and reimbursed clinical pharmacy services led mainly through legislative and accreditation changes.

With the commencement of healthcare facilities in CZ in 2009, hospitals were searching for clinical pharmacists (an obligatory legislative condition to ensure medication safety in all healthcare facilities) and some of these hospitals allowed clinical pharmacists to establish Departments of Clinical Pharmacy, independent of hospital pharmacies, functioning only as clinical workplaces and employing full-time specialists in clinical pharmacy. The first of such workplaces were founded in Hospital Na Bulovce and Hospital Na Homolce in Prague, the Czech Republic (Gregorová et al., 2014). In 2011, legislation defined special care by clinical pharmacists, called “clinical-pharmaceutical care (Czech Republic Parliament, 2011), which was a chievement in developing the whole effort of clinical pharmacy and remuneration of CPS in CZ.

This “special care by clinical pharmacists” helped to distinguish between the activities of specialists in clinical pharmacy from dispensing and consultation services in pharmacies and led to the extremely rapid development of further legislation, reimbursement schemes and new positions of clinical pharmacists as full-time experts in clinical pharmacy - specialists in acute care (since 2012) and ambulatory care (since 2021) (Ministry of Health of Czech Republic, 2012). An important point for establishing “clinical-pharmaceutical care” was an understanding that the key functions of clinical pharmacists are clinical, provided in or for clinical departments (not in and for pharmacies) and need a similar legislative framework as other clinical professions. In 2012, a decree ensured the availability of clinical pharmacists in inpatient wards (every hospital was obliged to use a minimum of 1 clinical pharmacist per 200 beds) (Ministry of Health of Czech Republic, 2012) and in 2015 reimbursed performance for the clinical pharmacy profession was approved by insurance agencies, first for acute care and then in 2020 for ambulatory care. Residential places (paid from funds of the Ministry of Health) are now also being discussed for clinical pharmacists.

All these changes led to a substantial increase is the number of specialists in clinical pharmacy in CZ (by 2022, 170 pharmacists graduated in specialty of clinical pharmacy and 150 are undersigned for the specialty training). Czech clinical pharmacists currently can be employed outside pharmacies in independent working positions (in acute and ambulatory care, and new positions are now established also in home care, long-term care facilities, hospices, etc.). Full-time clinical pharmacy positions enable clinical pharmacists in CZ to devote their professional time only and fully to comprehensive clinical medication reviews. They are involved also in other inpatient and outpatient activities ensuring medication safety and appropriate medication use (works on clinical guidelines, internal directives, appropriate medication use procedures, adherence support, etc.) (Gregorová et al., 2014; Vlček et al., 2016; Gregorová and Langmaierová, 2022).

In conclusion, after decades of unsuccessful “bottom-up efforts” only, contending with numerous barriers to the development of clinical pharmacy services in CZ (1975–2010), an important legislative change enabled change and thus turned the whole situation into the high demand for acute and primary care clinical pharmacists and fast development of CPS within the healthcare system.

Concerning a rapidly ageing population, CZ postgraduate education includes also the course for pharmacists in “Pharmaceutical care for geriatric patients,” focusing on the training of pharmacists firstly in simple medication reviews in older patients and preparing specialists for such signal reviews (providing in the future first medication checks, e.g., in social homes, nursing home facilities, etc.). These professionals will help to prioritize difficult cases of older patients for interventions of clinical pharmacists and to resolve simple medication-related problems with specific use of knowledge of geriatrics. This novel-certified course, focusing mainly on complex older adults with polypharmacy, is under the responsibility of clinical pharmacy education as well (Gregorová and Langmaierová, 2022).

In addition to advanced undergraduate, specialty and postgraduate (PhD) education, CP development in CZ has secured several important professional and legislative bodies, namely, the Czech Professional Society of Clinical Pharmacy and Section of Clinical Pharmacy of the Czech Pharmaceutical Society, Accreditation Committee of the Clinical Pharmacy of the Ministry of Health, and also Association of Clinical Pharmacy Workplaces (newly established in January 2022). CP in CZ has developed as a full educational, practical and scientific field (Gregorová et al., 2014; Vlček et al., 2016; Gregorová and Langmaierová, 2022).

#### 2.2.2 Slovenia

Slovenia developed advanced clinical pharmacy practice over the last 10 years, primarily due to highly-skilled and enthusiastic clinical pharmacists, support from the national insurance company and the Ministry of Health, as well as the shortage of clinical pharmacologists, geriatricians and other specialists (Slovenian Pharmacy Act, 2016; Stuhec et al., 2019; Stuhec, 2021; Stuhec and Tement, 2021). There is only one pharmacy school in Slovenia (Faculty of Pharmacy Ljubljana, University of Ljubljana) which provides undergraduate and postgraduate education. Specialization in clinical pharmacy is available and lasts 3 years. Clinical pharmacists are included within the two different Slovenian associations: The section of Clinical Pharmacists (organized as one of the sections of the Slovenian Pharmaceutical Society) and the Section of Hospitals Pharmacists (organized as one of the sections of the Slovenian Chamber of Pharmacy).

Three advanced and fully reimbursed clinical pharmacy services have been developed in Slovenia recently: clinical pharmacist consultant in all primary care settings from 2018, a seamless care system in all Slovenian hospitals from 2023 and a clinical pharmacist as a mandatory team member in the psychogeriatric team in all psychiatric hospitals from 2020 (Slovenian Pharmacy Act, 2016; Rules on the provision of pharmacy services by hospital pharmacies, 2022; Stuhec, 2021). All services require clinical pharmacy specialists and health institutions are paid extra for these services (e.g., hospitals and primary care settings). Clinical pharmacy is included and well-defined in Slovenia in the new Slovenian Pharmacy Act (valid from 2017). According to this Act, clinical pharmacy is an integral part of hospital pharmacy and pharmacists must be included in all medication-related processes and healthcare teams on the hospital ward. Each Slovenian hospital must provide clinical pharmacy services to its patients and hospital managers are responsible in ensuring the correct staffing level. In addition, a medication review service and many important clinical pharmacy services are defined and included in this Act (e.g., medication review, hospital clinical pharmaceutical services and seamless care) (Slovenian Pharmacy Act, 2016). The Slovenian Ministry of Health also developed an essential legislation document—Rules on the provision of pharmacy services by hospital pharmacies, where it is specified that only clinical pharmacists specialists (specialization is necessary) can work as clinical pharmacists independently in the hospital wards with beginning of year 2023 (Rules on the provision of pharmacy services by hospital pharmacies, 2022). Clinical pharmacists must be included in the team, have full access to patients and all datasets, and provide medication reviews where they decide. All seamless care processes have been defined inside this Sub Act (e.g., best possible medication history, medication reconciliation at admission, medication reconciliation at discharge, personal medication card before discharge and home dispensing).

A clinical pharmacy service cannot be developed without successful reimbursement models. In this context, all three models described have been reimbursed and some others are underway (e.g., outpatient clinics for patients with mental disorders in psychiatric hospitals). Clinical pharmacy ambulatory settings in all psychiatric hospitals (outpatients only for patients with mental disorders) have also been positively evaluated by the Slovenian Ministry of Health (evaluation committee) and proposed for national reimbursement in 2024. All psychiatrists in Slovenia will have an opportunity to refer patients with mental disorders to these settings in psychiatric hospitals, where clinical pharmacists specialists with experiences in mental disorders pharmacotherapy (minimum 3 years) will provide a medication review (Slovenian Ministry of Health, 2022). In 2023, discussions are taking place about pharmacist dependent prescriber in primary care settings (similar to the US model of collaborative practice agreement).

The Slovenian experience shows that clinical pharmacists must be actively included in reimbursement negotiations, health politics, health policy development, health insurance and the Ministry of Health (Stuhec, 2021).

### 2.3 Lack of CPS in most CEE countries

The Czech Republic and Slovenia have been able to fully implement and reimburse some CPS over recent years. Unfortunately, this is not the case for most of the other CEE countries and some other parts of Europe. The most recent situation of clinical pharmacy profession in 11 countries with a short description and summary of data are presented in Table 2.

**TABLE 2:** Summary of data about details of CPS in CEE countries (data valid as per January 2023).

Country	Professional education in clinical pharmacy included in		Hospital-based clinical pharmacy services	Outpatient clinical pharmacy services*	National association(s) of clinical pharmacy	Specialization, duration of the training
	Undergraduate training	Postgraduate training				
Austria	No	Yes	Yes Reimbursed	N/A	N/A	N/A
Entity of Bosnia and Herzegovina - Republic of Srpska	Yes, but optional	Yes, 3 years	Available in two hospitals Reimbursed	Available in selected places, Not reimbursed	N/A	Yes, 3 years
Bulgaria	Yes	Yes	Yes Not reimbursed	N/A	N/A	Yes, 3 years
Croatia	Yes	Yes	N/A	N/A	Section for clinical pharmacy under Croatian pharmaceutical society as well as the committee for clinical pharmacy under Croatian pharmaceutical chamber	Yes
Czechia	Yes	Yes	Yes Reimbursed	Yes Reimbursed	Section of Clinical Pharmacy of the Czech Pharmaceutical Society and Czech Professional Society of Clinical Pharmacy	Yes, 5 years
Estonia	Yes	Yes	N/A	N/A	Not available, existing within Estonian Society of Hospital Pharmacists	N/A
Greece	No	Yes	N/A	N/A	N/A	N/A
Hungary	Yes	Yes	Yes Not reimbursed	N/A	Hungarian Society of Hospital Pharmacist	Yes 3 years
					Hungarian Chamber of Pharmacists (hospital and clinical pharmacy section)	
					National Healthcare Service Centre (Hospital and clinical pharmacy section)	
Latvia	Yes	Yes, 2.5 years	Available in 5 hospitals Not reimbursed	N/A	Section at the Pharmacists’ Society of Latvia	Yes, 2.5 years
Poland	Yes	Yes	Yes, only in a few hospitals Not reimbursed	N/A	Yes, Polish Society of Clinical Pharmacy	Yes, 3 years
Serbia	Yes	No	Yes, only in a few hospitals Not reimbursed	Yes, only in a few hospitals Not reimbursed	The Section of Hospital pharmacists and Section of Pharmaceutical Care within the Pharmaceutical Association of Serbia	Yes, 3 years
Slovakia	Yes	Yes	Available Reimbursement for TDM laboratory items only	Available on demand. Not reimbursement	Section of Clinical Pharmacy, Slovak Pharmaceutical Association	Yes, 3 years
Slovenia	Yes	Yes	Yes Reimbursed	Yes Reimbursed	The Section of Clinical Pharmacists, which is organised as one of the sections of the Slovenian Pharmaceutical Society	Yes 3 years

N/A, not available; *includes services provided in general practice, outpatient clinics.

#### 2.3.1 Austria

Undergraduate CP lectures in Austria are available at the Universities of Innsbruck, Graz, Vienna and Salzburg. Currently, only basic elements of clinical pharmacy are scheduled in their curricula (Karl-Franzens-Universität Graz, 2018; Universität Wien, 2019; Paracelsus Medizinische Privatuniversität, 2020; Leopold-Franzens-Universität Innsbruck, 2021). The Austrian Chamber of Pharmacists offers a postgraduate weekend seminar focusing on basic medication analysis and clinical pharmacy service provision in community settings (Medikationsanalyse Basiskurs, 2023). The University of Vienna offers a postgraduate certificate course in clinical pharmacy which lasts one semester part-time (Klinische Pharmazie, 2012). There is currently no Master’s degree in clinical pharmacy offered at Austrian higher schools. However, it is planned to be implemented in autumn 2023. Only 42 of all 266 hospitals in Austria have a pharmacy department and even less offer the provision of clinical pharmacy services (Österreichische Apothekerkammer, 2020).

It is stated in the Austrian regulations for pharmacy services that “patient-oriented services (clinical pharmacy)” should be provided by the hospital pharmacy (RIS, 2022). Nevertheless, the provision of clinical pharmacy services is not included in the Austrian pharmacy profession act (RIS - Apothekengesetz - Bundesrecht konsolidiert, 2022). In the Austrian health structure plan (ÖSG) it is stated that “the availability of a sufficient number of pharmacists to provide clinical pharmaceutical services (including structured medication management, reduction of polypharmacy) must be ensured” (Bundes-Zielsteuerungskommission, 2017). However, there is no defined number as to how many clinical pharmacists per patient can be considered “sufficient.” Since January 2020 clinical pharmacy services can be registered in Austrian hospitals in the insurance reimbursement catalogues, although currently they are not directly remunerated and data is collected for informational reasons and for consideration of potential reimbursement implementation in the future. They must be requested by a physician or otherwise justified using standard operating procedures which are not readily available at this point and have to be implemented by each hospital individually (Müller, 2012). No national association of clinical pharmacy has been established. Austria has two associations of hospital pharmacists. In one of them membership is dependent on additional membership with the Austrian association of employed pharmacists (AAHP, 2023; VAAOE, 2023).

#### 2.3.2 Entity of Bosnia and Hercegovina—Republic of Srpska

In the Republic of Srpska, clinical pharmacy education has been included in undergraduate studies as an optional course. Starting from 2023/2024 academic year, CP will become an obligatory course and will include practical education at the hospital in addition to theoretical education at the university. A postgraduate course in clinical pharmacy was first introduced in the academic year 2021/2022, as a 3 years long specialization which led to the achievement of the title of a clinical pharmacist. Postgraduate studies at the Faculty of Medicine leading to a PhD degree are available for pharmacist with an option to choose a thesis topic from the field of clinical pharmacy.

Hospital-based CPS are implemented only in 2 out of 10 hospitals in the Republic of Srpska while outpatient clinical pharmacy services have not been developed yet. Clinical pharmacy is not included in the legislation, but there is official reimbursement for hospital-based CPS by the national health insurance fund (each written consultation of a clinical pharmacist is paid 20 euros). Standards for providing CPS are still not yet developed. There is no national association of clinical pharmacy in the Republic of Srpska due to a small number of practising clinical pharmacists. However, it is worth noting that, they all cooperate and exchange knowledge and experience on daily basis.

#### 2.3.3 Bulgaria

The legislative analysis showed that clinical pharmacy and clinical pharmacists are mentioned only in 2 documents: Ordinance 28 for the structure, order, and organization of work in the pharmacies (2008) and Good Pharmaceutical Practice in Bulgaria (2020). At least one Master of Pharmacy with a postgraduate degree in Clinical Pharmacy or an undergraduate specialization in Clinical Pharmacy should work in hospitals with more than 400 beds for active treatment or with at least 10 clinics/wards with beds, as well as in medical establishments that perform activities in medical oncology and/or clinical haematology. Clinical pharmacists should follow the principles of clinical pharmacy and be active participants in the treatment process.

The legislation in the country gives some basic rules as no specific clinical pharmacy services have been implemented for any group of Bulgarian patients. Despite the slow but stable progress in legislation on Clinical pharmacy, there are no specific clinical pharmacy services for patients nor reimbursement of such services. No national association of clinical pharmacy is established so far. However, there is a national non-profit organization, representing hospital pharmacists, offering clinical pharmacy training (OHPB, 2022). The organization has adopted the European Standards of hospital pharmacy with Sections 4, 5 detailing the responsibilities of clinical pharmacists (European Society of Hospital Pharmacy, 2023).

#### 2.3.4 Croatia

Although clinical pharmacy in Croatia has been developing since 1998, there are still no CPS included in the legislation. Clinical pharmacy education has a continuum through undergraduate and postgraduate education as well as clinical pharmacy specialization. After finishing the postgraduate degree in clinical pharmacy pharmacists can finish a full 3-year specialization in clinical pharmacy. Candidates become clinical pharmacists for primary or hospital care via two different specialization programs.

At the moment there are over 300 pharmacists with a postgraduate degree in clinical pharmacy as well as 40 specialists in the field of clinical pharmacy in Croatia. Regardless of the legislation gap CPSs and related activities are being developed by academic research and pilot projects in primary and hospital settings. In primary care, data from these activities show services having a positive impact on clinical, safety and quality of life (QoL) in anticoagulated patients through a randomized trial (Falamić et al., 2018; 2019; 2021). Other pilot projects providing comprehensive medication management have shown that the service is affordable and impacts positively on QoL, adverse drug reactions and healthcare utilization (Brajković et al., 2022b; 2022a; Mucalo et al., 2022). Medication reconciliation research from hospital setting shows clinical pharmacists detect medication discrepancies and prevent adverse patient outcomes at admission, as well as help reduce unintentional discrepancies associated with potential harm at discharge (Marinović et al., 2016; 2021). Clinical and hospital pharmacists in Croatia have proven that their involvement in the hospital medicines policy leads to substantial cost savings for the healthcare system (Javor et al., 2021).

The legislative gap regarding CPS is foreseen to be resolved in 2023 with the planned passing of the new Pharmacy Law. This should set up foundations for the negotiations of the CPS reimbursement. Standards of providing CPS and other activities of clinical pharmacists are yet to be developed on the national level, taking into account the practice and standards of other European countries and professional organizations*.*

#### 2.3.5 Estonia

Currently clinical pharmacy is included in the pharmacy curriculum at the University of Tartu as a separate subject. During the pharmacy internship, students can learn about the work of a clinical pharmacist in a hospital setting and select research project in the field of clinical pharmacy. An international e-learning clinical pharmacy and patient consultation continuous professional development course was developed at the same university as a form of postgraduate education for pharmacists who work either at hospitals or community pharmacies. The programme is characterized by a problem-based approach and a large amount of practical training (Clinical, 2023). An international MSc e-course in clinical pharmacy is under development. The listed courses are taught and/or developed by practicing clinical pharmacists (of whom there are less than 10 in Estonia) and who have acquired their education abroad, mainly in the United Kingdom and Ireland.

CPS are not currently standardized for neither hospital nor outpatient setting. Services are not reimbursed. They are provided in the hospital setting with a focus on the selection of the most suitable evidence-based treatment regimen for the patient and the achievement of the best treatment results. Medication review has been recently described as a structured clinical pharmacy service for primary care, but its implementation and reimbursement are still under development. Legalization of the clinical pharmacist specialty as a separate independent profession has been initiated. Clinical pharmacists belong to the Estonian Society of Hospital Pharmacists.

#### 2.3.6 Greece

Greece has three undergraduate schools of pharmacy all of which are located in large urban areas. Undergraduate studies consist of 4 years of theoretical and practical courses plus 1 year of traineeship at a hospital and a community setting. None of the three schools currently offer patient-related courses like pharmacotherapy or medicines optimization (Aristotle University of Thessaloniki, 2018). One of the three schools offers a postgraduate master’s degree in Clinical Pharmacy. It provides theoretical knowledge around disease management and includes a 5-month practical training at the hospital (Master in Clinical Pharmacy, 2023). Besides management tasks like stock turnover, online medicine orders or communicating with medicine suppliers both hospital and community pharmacists’ activities are limited mainly to the dispensing process (Greece Pharmacy Profession, 2023).

Pharmacists perform clinical activities in their daily practice only in a few private hospitals. The services offered mostly arise from the personal interest and engagement of the pharmacist rather than an organized framework. Community pharmacy owners belong to local pharmaceutical associations while pharmacists working in public hospitals are represented by the Panhellenic Association of Hospital Pharmacists or PEFNI (PEFNI, 1988). The former associations comprise the Panhellenic Association of Pharmacists or PFS (PFS, 2016). There is no association of clinical pharmacy at the moment in Greece. New legislation regarding secondary care has been introduced by the Greek government recently. Certain major changes have been implemented with the aim of improving the services offered by public hospitals, but pharmacists are not mentioned as providers of clinical services (Greek hospital legislation, 2023). Clinical tasks of community pharmacists are also limited and sporadic. Annual influenza vaccination has been incorporated in pharmacists’ activities in the last few years and further discussions are in progress with the view of being remunerated by the state.

#### 2.3.7 Hungary

In Hungary there are four Universities with a Faculty of Pharmacy. At each university students can learn about the concept of clinical pharmacy services. The University of Szeged has the longest history of undergraduate training for professional education in clinical pharmacy. According to the main European trends, Hungarian universities are willing to keep focusing on clinical pharmacy services. A 1-month hospital pharmacy internship is mandatory for every student before graduation. Postgraduate training is also available at all the universities, but it is not focused solely on clinical pharmacy and includes both hospital and clinical pharmacy training within 3-year study programme. After graduating in this specialization there are several 2-year sub-specialization opportunities (oncology, infectious diseases, paediatrics, medication information and counselling, parenteral medications, toxicology, clinical radiopharmacy), aimed at pharmacists working in a given area to gain relevant expertise. Postgraduate training can either be a state-run central residency training programme or self-financed, the former being the most common route.

Currently there are 501 hospital and clinical pharmacy specialists, 29 of them having additional sub-specialties. CPS are not yet reimbursed in Hungary. Good practice guidelines by the National Institute of Pharmacy and Nutrition exist but are limited to inpatient counselling by pharmacists, individual, per-patient drug dispensing (unit dose or daily dose), cytotoxic compounding, in-house parenteral infusion manufacturing (including parenteral nutrition therapy). More advanced CPS, including medication review are rare, but there are hospitals where it is achieved on specific wards. Partially as a result of the state-run central residency training programme, the number of pharmacists working in hospitals has been rapidly growing. The implementation of automated dispensing systems in Hungarian hospitals is also on the rise and there is a tendency for CPS to develop more effectively in these hospitals.

The last update of national pharmacy standards was made in 2012, but a new version is now being prepared. It is planned that it will define “clinical pharmacy services” and describe CPS processes. Three institutions are involved in matters relevant to clinical pharmacy in Hungary: Hungarian Society of Hospital Pharmacists, Hungarian Chamber of Pharmacists (section of hospital and clinical pharmacy), National Directorate General for Hospitals (Health Professional Colleges—Hospital and Clinical Pharmacy Council).

#### 2.3.8 Latvia

Both undergraduate and postgraduate education in clinical pharmacy are provided by Riga Stradins University. Undergraduate education - an introductory course in Clinical pharmacy is also provided by the University of Latvia (obligatory course). The first clinical pharmacist started working at Childrens’ Clinical University Hospital in October 2008.

Although there are many pharmacists with a Master level education in clinical pharmacy, only some of them work as clinical pharmacists. There are still very few hospital-based CPS. In total, four clinical pharmacists are actively working in five hospitals. One of the pharmacists works in two hospitals. There are no outpatient CPS at the moment. CPS are included in legislation but there is no special reimbursement for the services. There are no standards of providing CPS at the national level. Activities depend on particular hospital needs. Clinical pharmacy section is a part of the Pharmacists’ Society of Latvia.

#### 2.3.9 Poland

The first initiatives concerning clinical pharmacy in Poland date back to 1970s. Nevertheless, pharmacist involvement in direct patient care was not adopted into practice for a long time (Bryla et al., 2020). In the last few years, an increase in the importance of the pharmacist’s role and gradual development of CPS has been observed. In 2020, a national consultant for clinical pharmacy was appointed followed by subsequent appointments of regional consultants. The Pharmacy Profession Act was published the same year. It includes the definition of clinical pharmacy services.

In 2021 the Ministry of Health (MoH) released a report describing pharmaceutical care in community pharmacies (Polish Ministry of Health, 2020). Medicines review service was piloted in community pharmacies. The publication of pilot results was anticipated at the time of preparing this manuscript.

Further steps were taken to describe CPS and in 2022 another report prepared by a working group established by the MoH was released. The report describes 5 main services: medicines reconciliation, medicines review, patient education, therapeutic drug monitoring, specialized medicines service (includes high-risk oncology therapies and other specialist treatments dispensed from hospitals). The concept described in the document assumes pharmacist’s involvement in all levels of care–from admission to discharge, including CPS in general practice and long-term care facilities (Polish Ministry of Health, 2022). However, currently there are no formal requirements to employ clinical pharmacists and CPS are not reimbursed. Most activities are undertaken by pharmacist working in hospital pharmacy who are motivated to devote part of their time to clinical duties. A few pieces of works have described potential roles for the pharmacist and their benefits or the pharmacist’s perspective of the patient’s safety, but only one reported outcomes from a clinical pharmacy service fully integrated into medical care (Urbańczyk et al., 2022).

Clinical pharmacy is included as a subject in undergraduate education. Additionally, pharmacist can complete a postgraduate specialization which lasts 3 years. Around 270 people have already graduated obtaining this title, but estimates are probably not accurate as the programme changed over years and not everyone has formally registered. In 2020 Polish Society of Clinical Pharmacy was established. The Society released the first Polish standard in clinical pharmacy (Medicines reconciliation) and more standards are currently being developed (Bryla et al., 2020; Polish Society of Clinical Pharmacy, 2021).

#### 2.3.10 Serbia

Clinical pharmacy has been an obligatory subject during undergraduate studies at Faculty of Pharmacy University of Belgrade since October 2006 (University of Belgrade, 2023). Specialization in clinical pharmacy lasts 3 years and there are currently approximately 35 specialists (Study programme on clinical, 1960). Moreover, similar content is offered within the specialisation in pharmacotherapy (3 years’ duration, approximately 25 specialists), Pharmaceutical care (18 months’ duration, approximately 550 specialists) and Pharmacotherapy in pharmacy practice (18 months’ duration, approximately 100 specialists). Research in clinical pharmacy in Serbia is on the rise mainly due to project tasks within the specialization thesis and collaboration with the university.

Significant progress in delivering clinical pharmacy/pharmaceutical care services by pharmacists in Serbia has been made in the past decade. Pharmacists in primary care have piloted the following pharmacist roles: diabetes counsellor, asthma consultant, antibiotic consultant, new medicines service and counsellor on the safe use of medicines during breastfeeding. However, in many community pharmacies, services are still limited to traditional pharmacy practices such as procurement of drugs, extemporaneous compounding, dispensing of prescriptions, and selling medicines. There are only 5 services recognized officially by the nomenclature of health services in primary care settings (only two related to clinical pharmacy) and approximately 30 services in secondary and tertiary health settings (only a few related to clinical pharmacy), Pharmacists are not paid based on the services provided (Rule book on the nomenclature of health, 2023a; Rule book on the nomenclature of health, 2023b).

A new document on nomenclature based on piloted services with confirmed evidence is planned to be adopted next year. Moreover, Good Pharmacy Practice paper which defines standards and guidelines enabling the provision of pharmaceutical services was published in the Official Gazette and it needs to be applied from the beginning of April 2023 (Good pharmacy practice, 2021).

#### 2.3.11 Slovakia

There are two pharmacy schools providing pharmacy studies in Slovakia: Comenius University in Bratislava and Faculty of Pharmacy and University of Veterinary Medicine and Pharmacy in Košice. There is an obligatory course of clinical pharmacology/pharmacy and pharmacotherapy included in the undergraduate curriculum (Švec and Kuželová, 2012). Other courses available pharmacists are an obligatory one-semester courses of Social Pharmacy and Pharmacoeconomics, Pharmaceutical care and an optional course on Hospital Pharmacy. Postgraduate doctoral education is provided by Comenius University in Bratislava, Faculty of Pharmacy in the scientific programme of clinical pharmacy (Comenius University in Bratislava, 2022a; Comenius University in Bratislava, 2022b). Postgraduate specialty education in clinical pharmacy is provided by Slovak Medical University in Bratislava. Pharmacists need to complete the specialty in retail pharmacy in the first stage (3 years), thereafter they can continue studying clinical pharmacy for 2 years to become a clinical pharmacist (Minimal standard for specialty study program in clinical pharmacy, 2010).

The level of development of hospital-based and outpatient CPS is low in Slovakia. In fact, there are 116 hospitals (general and specialized), and only 16 clinical pharmacists’ posts. At present, 3 clinical pharmacists work full-time in departments of clinical pharmacology, together with clinical pharmacologists with the main interest in optimizing pharmacotherapy in hospitalized patients, therapeutic drug monitoring, identifying adverse drug reactions and interactions, administering medication via enteral feeding tube, medication risk assessment in pregnancy and lactation, patient education. Three clinical pharmacists are providing toxicological consultancy at the National Toxicological Information Centre. The others are based in hospital and community pharmacies. They are focusing on hospital/community pharmacy services and from the point of clinical pharmacy mainly in a medication information service.

CPSs are not mentioned within the Slovak legislation, but the Ministry of Health issued the document—Concept of healthcare in clinical pharmacy, which presents the standard of providing CPSs (Minimal standard for specialty study program in clinical pharmacy, 2010). CPSs are included in the catalogue of medical services, but are not reimbursed from compulsory insurance in Slovakia despite the efforts of the Section of clinical pharmacist. CP are associated in the Section of clinical pharmacy established in 1990 (Clinical Pharmacy Section, 2023).

## 3 Actionable recommendations

Due to underdevelopment of CPS in CEE countries we call for the undertaking of the necessary steps to allow its wide implementation. The most needed actions for the next decade are composite activities including supporting education and practice of clinical pharmacists with legislation and reimbursement of CPS. With this in place, one can expect optimized drug utilization, drug safety and medication adherence and consequently improved quality and cost-effectiveness of healthcare services in CEE countries. In light of these aims, following actionable recommendations can be set:

1. Including clinical pharmacy in the education portfolio in CEE countries both in undergraduate and postgraduate pharmaceutical education with the concomitant assurance of the possibility to perform clinical activities.
2. Enabling and supporting research in clinical pharmacy field, taking particular care of benchmarking of available interventions. Key clinical pharmacy institutions should be identified and collaborative programme needs to be developed.
3. Allowing interprofessional collaboration and adopting CPS across all healthcare settings with the aim to assure a high quality continuum of care.
4. Adopting best practices from other countries and guidance provided by relevant organisations and authorities, such as WHO or EDQM.
5. Creating national legislative framework supporting clearly defined workplaces for pharmacists and reimbursement schemes of CPS.
6. Forming national CP scientific societies to promote policy changes as a professional body. Working collaboratively with them to use their expertise and facilitate development of standards regarding clinical pharmacy profession and practice in CEE.
7. Campaigns directed to the public should be organized to promote and explain the role of clinical pharmacists in direct patient care.

## 4 Discussion

CEE countries have multiple challenges to face such as an ageing society with high prevalence of polypharmacy, shortage of healthcare professionals to assure continuity of care, financial constraints. At the same time, they are under great pressure to provide both high-quality and cost-effective health services. However, it does not differ from other European countries. Under such circumstances, it is reasonable to consider a wider engagement of clinical pharmacists who are well positioned to prevent and manage drug-related problems as well as ensuring safe and effective use of medications.

As described earlier, most of the CEE countries have not yet developed CPS to significant extent. Some steps have been made, but only in relation to improvements in areas of education and practice in order to implement clinical pharmacy services. In contrast, the Czech Republic and Slovenia belonging to CEE region have managed to successfully adopt solutions already existing in other countries with a long history of CPS presence. Consequently, CPS are currently available there in both hospitals and primary care. In both of these countries, the pharmacist’s efforts to develop appropriate skills and raise the awareness of benefits of CPS have resulted in important changes in legislation, which has paved the way to the reimbursement of CPS. Being inspired by these success stories, the following are important points to consider in developing CPS in the other CEE countries.

### 4.1 Education and research

The provision of high-quality CPS requires appropriate training. Therefore, continuous improvement of education (both undergraduate and postgraduate) and gaining relevant clinical skills in practice are of utmost importance. Most CEE countries have undertaken steps to include clinical pharmacy in their educational portfolio, but a lack of opportunities to practice in inpatient and outpatient settings can be an obstacle in achieving further progress. Pharmacists should have opportunities to practice in a clinical setting under the supervision of more experienced colleagues, similarly to other professions. This was also confirmed by a recent initiative led by the European Society of Clinical Pharmacy Education Committee, which aimed to map clinical pharmacy education and practice in Europe. Results of a cross-sectional survey focused on three complementary domains of undergraduate education, postgraduate education, and practice proved that whilst almost all out of 40 studied countries provided clinical pharmacy education at the undergraduate level, the breadth and depth varied. Around two-thirds of countries provided master level clinical pharmacy programmes which were also highly variable, with similar results for continuous professional development. These results prove that there is a need for further development of clinical pharmacy education at all levels (Moura et al., 2022). In addition, all CEE countries will be in need of academic teachers who are skilled both in research and practice.

Education is inseparably connected with research. Combining practice with conducting research builds up the evidence enabling the wider promotion of CPS. Therefore, activities of this kind should be encouraged in CEE countries in order to collect data on clinical pharmacy performance in different scenarios. Of note is the fact that comparing the data between the countries might be challenging, because of major differences in healthcare systems. However, it is entirely feasible to adapt such work to local systems. There is already an evidence base demonstrating that CPS are beneficial in multiple aspects such as optimized drug utilization and overall hospitalization costs (McMullin et al., 1999; Reardon et al., 2015; Rychlickova et al., 2016), increased drug safety (Anderson and Schumock, 2009; Simoens et al., 2011), improved medication adherence (Schulz et al., 2019; McCarthy and Thomas Bateman, 2022), reduced hospital admissions (Scullin et al., 2007; Gillespie et al., 2009), reduced length of hospital stay (Scullin et al., 2007), reduced outpatient healthcare utilization and costs (Byrne and Dalton, 2017), higher patient satisfaction (Yuliandani et al., 2022) and positive feedback from other healthcare professionals (Chevalier et al., 2016). Nevertheless, research on the theory and practice of CPS in CEE countries is still of value, as this may help to benchmark available solutions, prioritizing the best ones, and their wider implementation. Therefore, CEE should promote clinical pharmacy research based on the impact of clinical pharmacists on different outcomes (i.e., reduced average length of stay in hospitals, reduced number of and longer time to readmissions, number of outpatients visits, optimized drug utilization, improved drug safety, reduced medication burden and healthcare costs, higher patient satisfaction and improvement in patient adherence to prescribed medicines), which is often necessary for successful national reimbursement.

### 4.2 Interprofessional collaboration and development of core CPS

According to a number of standards, pharmacists should be members of a multidisciplinary team and use their expertise to optimize pharmacotherapy for individual patients (Bond et al., 2004; Mccusker et al., 2013; Fernandes et al., 2015). Collaborative, effective work as a team should be a starting point for other activities to ease the adoption of a new collaborative model. The duties of a clinical pharmacists can overlap with other professionals, but they complement each other. However, tasks and models of providing services together with shared responsibility need to be clearly identified. This helps to place CPS in the system.

Using experience from various countries, a number of evidence-based services could be implemented in CEE. This includes but is not limited to multidisciplinary ward rounds, medicines reconciliation, medicines review, patient education, medicines information, therapeutic drug monitoring, and pharmaceutical care plans (Bond et al., 2004; Mccusker et al., 2013; Fernandes et al., 2015). Additionally, services specific to a particular country could be adapted depending on the need. Currently, the CEE countries have managed to develop, to different extents, activities provided mainly in hospitals. However, the goal should be to cover both hospital and ambulatory care settings, including primary care and long-term care facilities as this also benefits patients and healthcare system (i.e., improved disease management and adherence) (Santschi et al., 2014). In order to assure patient-centeredness at all levels of the continuum of care, the models of “seamless care” or “integrated medicines management” should be implemented (Spehar et al., 2005; Scullin et al., 2007). Particular services can be implemented gradually, giving priority to the most strategic ones from the country’s perspective. National ministries of health, insurance companies, professional bodies, academia, scientific societies and patient organizations should be engaged in the process.

### 4.3 Legislative frameworks

All CEE countries have demonstrated a substantial “bottom-up” approaches in developing CPS in their regions. Unfortunately, in many cases, years of efforts did not allow them to reach the level of countries recognized as models of best practice. Analyzing available data, it seems that they mainly lack appropriate legislative frameworks to support the shift from medicine supply roles to pharmacist delivered patient-centered services for the purpose of improving rational use of medications. Stakeholders need to acknowledge that national healthcare systems cannot afford not to use the full potential of pharmacists. Therefore, CPS should no longer be optional (because then in essence they would be excluded from the team and patients), but should be widely adopted similarly to other evidence-based solutions. This requires appropriate legislation and setting up of framework for new services using experience from other countries. It should also include constituting workplaces for clinical pharmacists in healthcare settings and aiming to reimburse services for wider adoption of process changes.

However, there is a real need for “top-down” activities. National policies and legislation need to support clinical pharmacy programs. Only with this support in place, will clinical pharmacists have the resources and support needed to provide high-quality, evidence-based care to patients. This also includes relevant funding for clinical pharmacy education, research, and practice, as well as changes in laws and regulations that allow clinical pharmacists to practice to the full extent of their training and expertise. In addition, residency spaces for CP should be secured and costs covered similarly to other medical professions.

As mentioned in the “Introduction” section, in March 2020 the Committee of Ministers of the Council of Europe adopted Resolution CM/Res(2020)3 on the implementation of pharmaceutical care for the benefit of patients and health services (Committee of Ministers Resolution, 2020). The Resolution and upcoming EDQM’s guidelines on Medication Review provide national authorities and healthcare professionals with guidance on how to implement pharmaceutical care and clinical pharmacy services, when needed through the allocation of appropriate budgetary resources such as incentives for performing pharmacy services or workforce resources.

Governments, competent health authorities and professional bodies across Europe are encouraged to put into practice the above resolution in their countries with a view to promote patient-centered care, encourage more responsible use of medicines, contribute to rationalizing healthcare resources, and helping reduce inequalities in healthcare in Europe.

Whenever possible, cooperation should be established with other international organizations and healthcare stakeholders to develop synergies, avoid duplication of efforts, and eventually meet the common objective of achieving better, patient-centered healthcare in Europe through policy-making decisions at the governmental level.

Another important document setting the scene for CPS in CEE is Medication without Harm, the third global patient safety challenge launched in 2017 by the WHO (WHO, 2017).

### 4.4 Stimulating role of European, national and international associations of clinical pharmacy

Not all CEE countries have established societies representing the clinical pharmacy profession which could advocate for the development of CPS and set standards of practice. However, existing national and International pharmacy professional societies and associations can support developments of CPS in CEE countries by forming powerful coalitions, harnessing expertise, facilitating learning and providing tools. Hence, an unprecedented role can be played by European Society of Clinical Pharmacy (ESCP), which was founded in 1979 and now has members from more than 45 countries across the globe, representing all pharmacy sectors. ESCP is an organization that promotes, supports, implements and advances education, practice and research in clinical pharmacy in order to optimize outcomes for patients and society (European Society of Clinical Pharmacy, 2023a). The vision of ESCP is to play the role of an international leader in advancing quality and innovation in clinical pharmacy education, practice and research. Several years ago, ESCP launched a ‘Best Practice Papers’ initiative in collaboration with the International Journal of Clinical Pharmacy. These papers aim to disseminate best practices in clinical pharmacy, and to enhance the exchange of knowledge and experiences to promote innovative and sustainable clinical pharmacy service. Best practices relate to developments in practice and education, which are supported by thorough development and implementation processes along with high quality, robust and rigorous research evidence of evaluation outcomes. These outcomes may include aspects such as acceptability, adoption, appropriateness, effectiveness, cost-effectiveness, efficiency, satisfaction, sustainability, etc. With a focus on development, implementation and evaluation, there is consideration of key facilitators, barriers and how these were overcome. It is hoped that these will act as a stimulus for similar developments in other countries, including CEE. In collaboration with the European Association of Hospital Pharmacists, ESCP launched the Oath to Society in October 2021. The Oath is intended to act as a contract for excellence in providing compassionate patient care, working as part of the healthcare team, advancing the pharmacy profession, and showcasing how pharmacists work every day. It also represents the promise made to patients and the public, and healthcare professionals and acts as a compass for the highest standards of ethics, integrity, and professionalism (European Society of Clinical Pharmacy, 2023b).

### 4.5 Need for public campaigns to increase the awareness of clinical pharmacy in CEE countries among healthcare professionals and lay public

Public campaigns are important to promote clinical pharmacy, because they raise awareness about the role and value of clinical pharmacists in healthcare. By educating the public and healthcare professionals about the expertise and services that clinical pharmacists provide, such as medication management, campaigns can help increase the demand for these services. Additionally, public campaigns can help to dispel any misconceptions about the role of clinical pharmacists and demonstrate the valuable contributions they make to the healthcare team. This in turn can help to increase the visibility and recognition of the profession.
